# Application of transvaginal color doppler ultrasound in early diagnosis of threatened abortion and its correlation with serum levels of β - hCG and CA125

**DOI:** 10.3389/fendo.2026.1773779

**Published:** 2026-07-15

**Authors:** Li Cao, Hongbing Xiang, Dongfang Ren, Jinsheng Li

**Affiliations:** 1 Department of Ultrasound Medicine, Nanyang Central Hospital, Nanyang, Henan, China; 2 Department of Laboratory, Nanyang Central Hospital, Nanyang, Henan, China

**Keywords:** CA-125 antigen, diagnosis, doppler ultrasonography, threatened abortion, β-chorionic gonadotropin

## Abstract

**Objective:**

To explore the value of transvaginal color Doppler ultrasound (TVCDS) combined with serum β-human chorionic gonadotropin (β-HCG) and carbohydrate antigen-125 (CA125) in diagnosing threatened abortion and predicting adverse pregnancy outcomes.

**Methods:**

A retrospective case-control study included 98 pregnant women with threatened abortion (observation group) and 100 normal pregnant women (control group). Serum indicators, corpus luteum pulsatility index (PI) and resistance index (RI) were compared. Correlations and combined predictive efficacy were analyzed using multivariate logistic regression and receiver operating characteristic (ROC) curves.

**Results:**

TVCDS showed 92.86% sensitivity, 100% specificity and 0.912 AUC in diagnosing threatened abortion. The observation group had higher corpus luteum PI, RI and CA125, but lower β-HCG than the control group (all P<0.001). Corpus luteum PI and RI were negatively correlated with β-HCG (r=-0.401, -0.465) and positively with CA125 (r=0.511, 0.492, all P<0.01). Multivariate logistic regression identified PI, β-HCG, and CA125 as independent predictors of failed pregnancy preservation. The combined predictor (predicted probability from the regression model) yielded an AUC of 0.931, superior to any single indicator (P<0.001).

**Conclusion:**

TVCDS combined with serum β-HCG and CA125 improves the efficacy in diagnosing threatened abortion and predicting adverse pregnancy outcomes. The multivariate model provides a reliable tool for individual risk assessment.

## 1 Introduction

A threatened abortion refers to minimal vaginal bleeding without the expulsion of pregnancy tissue before 28 weeks of gestation, with risk of progressing to inevitable or missed abortion in the absence of timely and effective intervention. It has a multifactorial pathogenesis that severely compromises women’s physical and mental health, with nearly 80% of miscarriages occurring during early pregnancy (1). Serum β-human chorionic gonadotropin (β-HCG) is a primary hormone secreted during early pregnancy that is essential to maintain pregnancy. Meanwhile, carbohydrate antigen-125 (CA-125) is a tumor antigen marker derived from the surface of coelomic epithelium, reproductive tract mucosa, and ovarian epithelial cells. According to prior report, there were elevated CA-125 levels in women experiencing impending miscarriage, indicating decidual cell damage and trophoblastic cell separation. Furthermore, ultrasonography has become a key tool in pregnancy assessment, with high reliability in monitoring normal and abnormal pregnancies as well as early embryonic development. It is important to emphasize that threatened abortion remains primarily a clinical diagnosis, and ultrasound parameters serve as adjunctive tools to identify patients at higher risk of adverse outcomes. Previous studies on threatened abortion have mainly focused on the simple combination of “ultrasound + serum indicators”, without in-depth analysis of the specific correlation between corpus luteum blood flow parameters and serum indicators, as well as discussions on application scenarios in grassroots hospitals (2). Clinically, there is a significant variation in the success rate of pregnancy preservation among threatened abortion patients. Indiscriminate pregnancy preservation may not only increase patients’ financial and psychological burdens, but also lead to the waste of medical resources, highlighting an urgent need for accurate predictive methods to forecast pregnancy preservation outcomes (3). Accordingly, this retrospective case-control study was performed to analyze the application of transvaginal color Doppler sonography (TVCDS) in early threatened abortion diagnosis and its correlation with serum β-HCG and CA125 levels. This study is expected to provide clinical guidance for early diagnosis and prediction of pregnancy preservation outcomes. The findings are reported as follows.

## 2 Methods

### 2.1 Participants

This retrospective case-control study was conducted with the inclusion of 98 threatened abortion patients (the observation group) and 100 normal pregnancy cases (the control group) who were admitted to our hospital from January 2020 to June 2022.

Diagnostic criteria: The diagnosis of threatened abortion was established according to the 9th edition of *Obstetrics and Gynecology* (People’s Medical Publishing House, China) (4), which defines threatened abortion as vaginal bleeding before 28 weeks of gestation without expulsion of pregnancy tissue, closed cervical os, and uterus size consistent with gestational age. TVCDS was used as an adjunctive tool to assess embryonic viability and corpus luteum function. The following ultrasound findings were recorded to support the diagnosis and risk assessment (5–7): ① Irregular gestational sac shape and low position; ② the presence of fluid sonolucent areas around the gestational sac; ③ corpus luteum blood flow parameters pulsatility index (PI) >0.80 or resistance index (RI) >0.60; and ④ the absence of embryonic bud and primitive cardiac tube pulsation (consistent with gestational week but not visible).

Inclusion criteria: The observation group: (1) Primiparous women with a single pregnancy at 6–8 weeks of gestation (confirmed by last menstrual period and gestational age determined by ultrasound); Primiparous women were selected to minimize confounding factors related to prior obstetric history, such as recurrent pregnancy loss or previous uterine instrumentation, which could independently affect pregnancy outcomes. (2) Diagnosis consistent with the criteria of threatened abortion in the 9th edition of *Obstetrics and Gynecology*: vaginal bleeding before 28 weeks of gestation without the expulsion of pregnancy tissue, uterus size matching gestational age, closed cervical os, intact fetal membranes, and symptoms relieved after rest and treatment (8); and (3) no prior treatment using progesterone, human chorionic gonadotropin (HCG), etc., for pregnancy preservation before admission.

The control group: ① Primiparous women with a single pregnancy at 6–8 weeks of gestation; ② no symptoms such as vaginal bleeding or abdominal pain; ③ normal embryonic development (visible gestational sac, embryonic bud and primitive cardiac tube pulsation) indicated by ultrasound; ④ β-HCG and CA-125 levels within the reference ranges of normal pregnancy; and ⑤ no pregnancy complications or comorbidities.

Exclusion criteria: (1) complicating diseases such as liver/kidney dysfunction, malignancies, blood disorders, and immune system diseases; (2) uterine or genital tract anomalies; (3) endometriosis or adenomyosis; (4) polycystic ovary syndrome diagnosed according to the 2013 Rotterdam criteria (the presence of at least two of the following three symptoms, with simultaneous exclusion of other hyperandrogenic conditions: ① oligo/anovulation; ② clinical and/or biochemical signs of hyperandrogenism; and ③ polycystic ovaries on ultrasound; thyroid dysfunction (abnormal thyroid stimulating hormone (TSH) levels: TSH >2.5 mIU/L or <0.1 mIU/L in early pregnancy) (9); (5) history of smoking or alcohol consumption; and (6) two or more previous miscarriages. This study was approved by the Ethics Committee of our hospital.

### 2.2 Methods

#### 2.2.1 Detection methods

Upon admission, all subjects were subjected to the collection of 4ml of venous blood from the elbow vein (between 9:00-11:00 am). The samples were centrifuged at 3,000 rpm for 10 minutes to obtain the supernatant for subsequent analysis within 2 hours. Serum levels of β-HCG (0.1–100000 mIU/mL, ADVIA Centaur β-HCG, Siemens) and CA125 (0–1000 IU/mL, ADVIA Centaur CA125II, Siemens) were detected via chemiluminescence using Hitachi 7600i Fully Automatic Biochemical Analyzer.

#### 2.2.2 TVCDS

TVCDS was performed by employing GE E10/E8 color Doppler ultrasound (probe frequency of 5-9 MHz) in primiparous women during their 6th and 8th weeks of pregnancy. In bladder emptying state, subjects were examined by inserting the vaginal probe gently into the vagina and placing against the posterior fornix. Multi-planar scanning was adopted to observe the uterus and bilateral adnexal regions. The corpus luteum blood flow was measured at the central artery of the corpus luteum, with three consecutive cardiac cycles averaged. Two experienced radiologists, each with >5 years of ultrasound diagnostic experience, independently performed all ultrasound measurements in a double-blind manner, with each reader blinded to the other’s measurements and to the clinical information of the participants. A random subset of 30 cases (approximately 15% of the total sample) was selected for inter-observer reproducibility analysis. The intraclass correlation coefficient (ICC) was used to assess consistency for continuous variables (PI and RI), while Cohen’s kappa coefficient was used for categorical diagnostic classifications. The uterine artery blood flow was measured at its origin, with any branching vessels avoided, using the same method as for the corpus luteum blood flow.

#### 2.2.3 Data collection

Baseline data: This study collected general information from the electronic medical record system in all patients upon hospital admission, with data verification. These baseline data included age, body mass index (BMI), duration of amenorrhea, TSH levels, history of abortion, etc.

Outcome Measures: ① Diagnostic indicators: β-HCG and CA125 levels, corpus luteum blood flow parameters PI and RI; ② Pregnancy preservation outcomes: All patients in the observation group received standardized pregnancy preservation treatment, and were followed up until week 12 of pregnancy. Adverse pregnancy outcomes were defined as inevitable abortion or missed abortion, while successful outcomes were defined as continued embryonic development without adverse events.

All data were entered into an Excel by two researchers independently. Cross-check for discrepancies was conducted after data entry. In addition, to ensure data accuracy, the original medical records were verified if discrepancies were found.

#### 2.2.4 Representative doppler imaging

**Figure 1:**
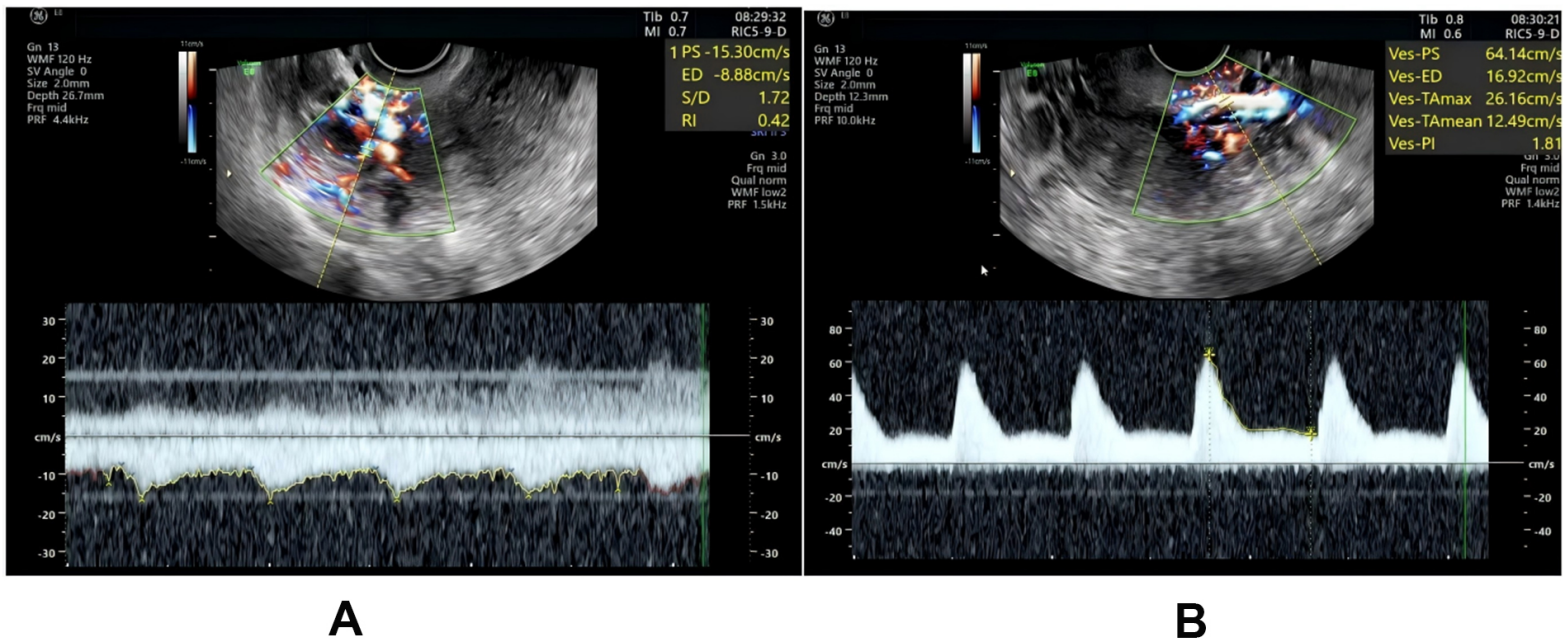
Representative transvaginal color doppler images. **(A)** Corpus luteum blood flow: spectral Doppler waveform showing hemodynamic parameters (e.g., RI = 0.42). **(B)** Right uterine artery blood flow: spectral waveform showing pulsatility index (PI = 1.81). These parameters were used to assess luteal perfusion and predict pregnancy outcomes.

Representative transvaginal color Doppler images of the corpus luteum are shown in **Figure 1**, demonstrating the typical vascularization pattern around the corpus luteum. Corresponding spectral Doppler waveforms are presented in **Figure 1**, illustrating the measurement of pulsatility index (PI) and resistance index (RI). These images exemplify the standard acquisition technique used in this study, with three consecutive cardiac cycles averaged for each measurement.

### 2.3 Statistical analysis

Data analysis was performed using SPSS 22.0. Normally and non-normally distributed data were expressed as mean ± SD or median (IQR), with t-test or Mann-Whitney U test as appropriate. Correlation analysis used Pearson or Spearman method. Inter-observer reproducibility was assessed using ICC and Cohen’s kappa. ROC curves were used to evaluate diagnostic and predictive values, with AUC, 95% CI, and Youden index calculated.

To identify independent predictors of adverse pregnancy outcomes, multivariate logistic regression with forward stepwise (likelihood ratio) selection was performed, including variables with P<0.05 in univariate analysis (PI, RI, β-HCG, CA125). The predicted probability (PRE_1) from the final model was saved and used as a combined indicator to construct a new ROC curve. The predictive performance of this combined probability was compared with single parameters using the DeLong test. A two-tailed P<0.05 was considered significant.

The predicted probability (PRE_1) of failed pregnancy preservation was calculated using the following logistic regression equation derived from the final multivariate model: PRE_1 = 1/(1+e^{-z}), where z is the linear combination of independent variables weighted by their respective regression coefficients (β). The cutoff value for PRE_1 was determined using the maximum Youden index from the ROC curve.

## 3 Results

### 3.1 Baseline data

Inter-observer reproducibility was assessed in a random subset of 30 cases (15.2% of the total sample). For corpus luteum blood flow parameters, the intraclass correlation coefficient (ICC) was 0.87 (95% CI: 0.82-0.91) for PI and 0.85 (95% CI: 0.79-0.89) for RI, indicating good to excellent consistency. For the categorical diagnosis of threatened abortion based on TVCDS findings, Cohen’s kappa coefficient was 0.89 (95% CI: 0.83-0.95), representing good agreement between the two radiologists.

Comparison of the clinical baseline data between the observation and control groups is shown in **Table 1**. There were no statistically significant differences in age, BMI, duration of amenorrhea, TSH levels, and history of abortion between the two groups (all *P>*0.05).

**Table 1:** Comparison of clinical baseline data between the observation and control groups.

Clinical indicators	Observation group (n=98)	Control group (n=100)	t/χ² value	P value
Age (years)	29.80 ± 4.11	30.01 ± 4.92	-0.326	0.745
Body mass index (kg/m²)	23.02 ± 2.05	22.95 ± 2.13	0.236	0.814
Duration of amenorrhea (days)	44.03 ± 5.82	43.89 ± 6.10	0.165	0.869
TSH(mIU/L)	1.85 ± 0.72	1.78 ± 0.69	0.689	0.492
History of abortion (cases, %)	5(5.10%)	4(4.00%)	0.152	0.697

### 3.2 Diagnostic value of TVCDS for threatened abortion

The sensitivity, specificity, accuracy, positive predictive value, and negative predictive value of TVCDS in diagnosing threatened abortion were 92.86% (91/98), 100.00% (100/100), 96.46% (191/198), 100.00% (91/91), and 93.46% (100/107), respectively. The AUC was 0.912 (95% CI: 0.875-0.949, *P* < 0.001).

### 3.3 Comparison TVCDS parameters between the observation and control groups of

As shown in **Table 2**, the corpus luteum blood flow parameters PI and RI values were significantly higher in the observation group than those in the control group (both *P* < 0.001). There were no significant differences in uterine artery blood flow parameters PI and RI values between groups (both *P>*0.05).

**Table 2:** Comparison of the corpus luteum blood flow parameters between observation and control groups.

Group	Number of cases	Corpus luteum		Uterine artery	
		PI	RI	PI	RI
Observation group	98	0.84 ± 0.07	0.65 ± 0.06	1.43 ± 0.07	0.79 ± 0.06
Control group	100	0.73 ± 0.09	0.52 ± 0.07	1.44 ± 0.06	0.80 ± 0.08
t		9.587	14.018	-1.080	-0.994
*P*		<0.001	<0.001	0.281	0.322

### 3.4 Comparison of serum β-HCG and CA125 levels between the observation and control groups

As presented in **Table 3**, compared to the control group, the observation group had significantly lower serum β-HCG levels, while obviously higher CA125 levels (both *P* < 0.001).

**Table 3:** Comparison of serum β-HCG and CA125 levels between the observation and control groups.

Group	Number of cases	HCG (mIU/ml)	CA125(IU/ml)
Observation group	98	12243.30 ± 210.32	40.54 ± 13.32
Control group	100	33104.54 ± 334.72	15.59 ± 3.40
t		-523.885	18.139
*P*		<0.001	<0.001

### 3.5 Correlation analysis

Shapiro-Wilk tests showed normal distribution of PI, RI, serum β-HCG, and CA125 (all *P* > 0.05). Pearson correlation analysis revealed that corpus luteum blood flow parameters PI and RI were significantly correlated with serum markers. Specifically, PI and RI were negatively correlated with β-HCG (r = -0.401 and -0.465, respectively, both *P* < 0.01) and positively correlated with CA125 (r = 0.511 and 0.492, respectively, both *P* < 0.01).

### 3.6 Comparison of TVCDS parameters and serum markers between successful and adverse pregnancy outcomes in the observation group

There were 30 cases of adverse pregnancy outcomes in the observation group. Among them, there were 15 cases (50.00%) of corpus luteum dysfunction, 8 cases (26.67%) of chromosomal abnormalities, 4 cases (13.33%) of uterine malformation, and 3 cases (10.00%) due to other reasons. Although uterine anomalies were listed as exclusion criteria at enrollment, these four cases were identified during follow-up imaging and were retained in the analysis to reflect real-world clinical scenarios and to avoid selection bias. Their exclusion would not have altered the overall statistical conclusions. Although uterine anomalies were listed as exclusion criteria at enrollment, these four cases were identified during follow-up imaging and were retained in the analysis to reflect real-world clinical scenarios and to avoid selection bias. Their exclusion would not have altered the overall statistical conclusions. According to the analysis in **Table 4**, women with adverse pregnancy outcomes had significantly higher corpus luteum blood flow parameters PI, RI, and CA125 levels (all *P* < 0.001), and lower HCG levels compared to those with successful preservation (*P* < 0.001).

**Table 4:** Comparison of TVCDS parameters, serum β-HCG, and CA125 between successful and adverse pregnancy outcomes groups.

Group	Number of cases	Corpus luteum		Uterine artery		HCG (mIU/ml)	CA125 (IU/ml)
		PI	RI	PI	RI		
Successful	68	0.80 ± 0.08	0.62 ± 0.07	1.44 ± 0.08	0.78 ± 0.07	13044.02 ± 305.59	37.03 ± 12.21
Failed	30	0.93 ± 0.07	0.72 ± 0.08	1.41 ± 0.09	0.81 ± 0.09	10428.33 ± 360.41	48.50 ± 13.01
t		-7.691	-6.236	1.646	-1.787	36.932	-4.201
*P*		<0.001	<0.001	0.103	0.077	<0.001	<0.001

### 3.7 Univariate and multivariate logistic regression for predicting adverse pregnancy outcomes

Univariate logistic regression was first performed to assess the individual association of each parameter with failed pregnancy preservation. As shown in **Table 5**, corpus luteum PI, RI, β-HCG, and CA125 were each significantly associated with adverse outcomes in univariate analysis (all *P* < 0.01). The AUC values for individual parameters ranged from 0.679 to 0.853, indicating modest discriminative ability.

**Table 5:** Univariate logistic regression results and individual ROC curve parameters.

Indicator	AUC (95% CI)	Youden index	Cutoff value	Sensitivity (%)	Specificity (%)	Univariate OR (95% CI)	P value
PI	0.843 (0.766–0.921)	0.624	>0.90	80.0	82.4	1.84 (1.32–2.56)	<0.001
RI	0.787 (0.680–0.893)	0.549	>0.70	66.7	88.2	1.45 (1.12–1.89)	0.004
β-HCG (per 1000 mIU/mL)	0.853 (0.762–0.944)	0.600	<11689.91	60.0	100.0	0.78 (0.68–0.89)	<0.001
CA125	0.679 (0.563–0.795)	0.291	>51.25	46.7	82.4	1.12 (1.05–1.20)	0.002

OR for β-HCG is per 1000 mIU/mL decrease (since lower values are associated with failure).

Cutoff values were determined based on the maximum Youden index from ROC analysis. PI, pulsatility index; RI, resistance index; β-HCG, beta-human chorionic gonadotropin; CA125, cancer antigen 125; AUC, area under the curve; CI, confidence interval.

Variables with P<0.05 in univariate analysis (PI, RI, β-HCG, CA125) were entered into multivariate logistic regression (forward stepwise LR). The results (**Table 6**) showed that PI, β-HCG, and CA125 were independent predictors of failed pregnancy preservation (all P<0.05), while RI was excluded due to collinearity with PI (r=0.73). The model showed good fit (Hosmer-Lemeshow χ²=5.62, P = 0.69; Nagelkerke R²=0.58).

**Table 6:** Multivariate logistic regression for predicting failed pregnancy preservation in threatened abortion.

Variable	β coefficient	Wald χ²	OR (95% CI)	P value
PI	2.145	8.92	8.55 (2.04–35.81)	0.003
β-HCG (per 1000 mIU/mL decrease)	-0.512	9.34	0.60 (0.44–0.82)	0.002
CA125	0.087	7.68	1.09 (1.03–1.16)	0.006
Constant	-9.324	12.56	–	<0.001

### 3.8 ROC analysis for predicting adverse outcomes (using predicted probability from the model)

**Figure 2:**
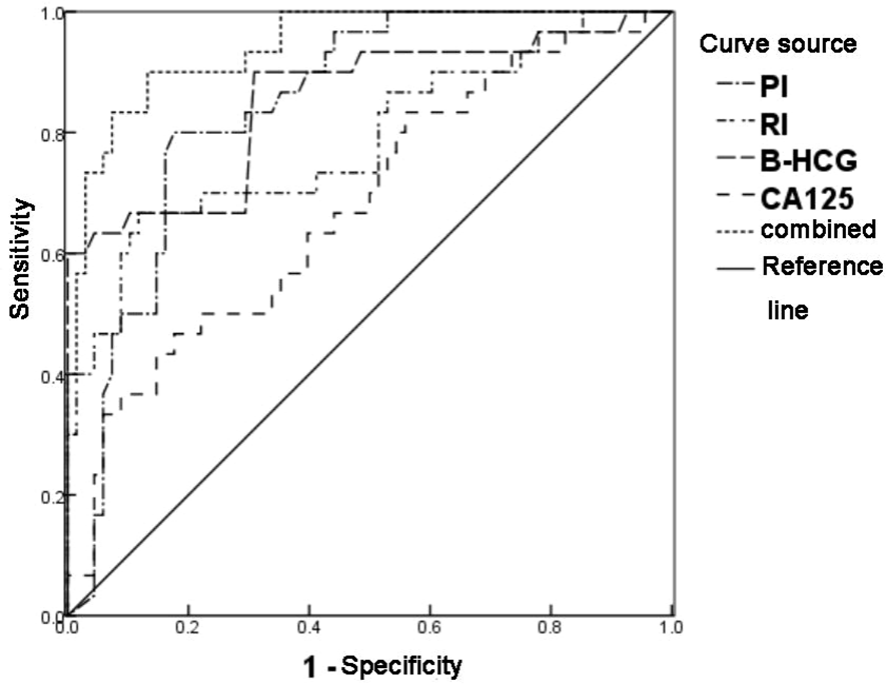
ROC curve.

The predicted probability (PRE_1) derived from the logistic regression model was used as a new combined indicator. ROC analysis (**Table 7**; **Figure 2**) showed that the AUC of the combined predicted probability was 0.931 (95% CI: 0.882–0.980), which was significantly higher than that of any single parameter (PI 0.843, RI 0.787, β-HCG 0.853, CA125 0.679; all P<0.01 by DeLong test). At the optimal cutoff of 0.68 (Youden index 0.749), the sensitivity and specificity were 86.7% and 88.2%, respectively. This performance was comparable to the original serial combination (AUC = 0.944, P = 0.48 for comparison), but the model-based probability offers continuous risk estimation and is more suitable for individualized decision-making.

**Table 7:** ROC analysis of single parameters and combined predicted probability for predicting failed pregnancy preservation.

Indicator	AUC	95% CI	Sensitivity (%)	Specificity (%)	Youden index	Cutoff
PI	0.843	0.766–0.921	80.0	82.4	0.624	0.90
RI	0.787	0.680–0.893	66.7	88.2	0.549	0.70
β-HCG (mIU/mL)	0.853	0.762–0.944	60.0	100.0	0.600	11689.91
CA125 (IU/mL)	0.679	0.563–0.795	46.7	82.4	0.291	51.25
**Combined predicted probability**	**0.931**	**0.882–0.980**	**86.7**	**88.2**	**0.749**	**0.68**

Bold values indicate the combined diagnostic results of four indicators.

The predicted probability (PRE_1) derived from the logistic regression model was used as a new combined indicator. Specifically, PRE_1 was calculated for each patient using the equation: 1/(1+e^{-z}), with z = -9.324 + 2.145×PI -0.512×(β-HCG per 1000 mIU/mL decrease) + 0.087×CA125. The optimal cutoff value for PRE_1 was 0.68, corresponding to the maximum Youden index of 0.749.

### 3.9 Sensitivity analysis

To test the robustness of our findings, several sensitivity analyses were performed:

1. Exclusion of uterine anomaly cases: Four patients in the observation group were later found to have uterine malformations (originally exclusion criteria but retained for real-world representation). After excluding these four cases, multivariate logistic regression still identified PI, β-HCG, and CA125 as independent predictors (ORs: 8.12, 0.61, 1.08 respectively; all P<0.05), and the AUC of the combined probability was 0.928, consistent with the primary analysis.
2. Alternative variable selection method (Lasso regression): Lasso regression selected the same three predictors (PI, β-HCG, CA125), supporting model stability.
3. Subgroup analysis by gestational age (6–7 weeks vs. >7–8 weeks): The AUC of the combined probability remained >0.90 in both subgroups, indicating good performance across early pregnancy stages.

These sensitivity analyses confirm that our conclusions are not biased by a few cases or the choice of statistical method.

## 4 Discussion

With China’s rapid economic and social development, women face increasing work pressure and delayed childbearing, making threatened abortion a prevalent concern in obstetrics and gynecology (10). The spontaneous miscarriage rate ranges from 15% to 40%, with over 75% occurring before the 16th week of gestation. Threatened abortion has a multifactorial pathogenesis, potentially involving chromosomal abnormalities, embryonic developmental defects, advanced maternal age, infections, and exposure to harmful chemicals. Currently, clinical prevention relies on avoiding overexertion, cautious medication use, minimizing radiation exposure, and maintaining a balanced diet (11, 12). However, indiscriminate pregnancy preservation not only wastes medical resources but also imposes financial and psychological burdens on patients. Therefore, there is an urgent need for accurate predictive tools to assess disease progression and prognosis early, enabling clinicians to guide individualized treatment and help patients—even those who ultimately experience failed preservation—better understand and accept their situation (13–16). In this context, TVCDS parameters serve as adjunctive tools to identify high-risk patients rather than replace clinical diagnosis (17).

In this study, the observation group had significantly lower serum β-HCG levels (12,243.30 ± 210.32 mIU/mL) and higher CA125 levels (40.54 ± 13.32 IU/mL) than the control group (both P<0.001). Similarly, among threatened abortion patients, those with adverse pregnancy outcomes showed even lower β-HCG (10,428.33 ± 360.41 mIU/mL) and higher CA125 (48.50 ± 13.01 IU/mL) than those with successful preservation (both P<0.001). β-HCG, secreted by placental syncytiotrophoblasts, reflects corpus luteum function and placental development; its insufficient rise indicates trophoblast dysfunction (19, 20). CA125, elevated in decidual cell damage, enters maternal circulation when the trophoblast separates from the decidua (18, 19). These findings support the combined use of β-HCG and CA125 in assessing threatened abortion prognosis.

According to recent reports, TVCDS can accurately assess fetal development during pregnancy, detecting intrauterine gestational sacs earlier than abdominal ultrasound (20). After implantation, trophoblastic cells invade and remodel uterine spiral arteries, leading to decreased resistance. However, in this study, uterine artery PI and RI showed no significant differences between the observation and control groups (both P>0.05), possibly due to the small sample size or the fact that uterine blood flow increases more notably with advancing gestational age (21–23). In contrast, the corpus luteum of pregnancy secretes progesterone essential for embryonic support. The observation group had significantly higher corpus luteum PI (0.84 ± 0.07 vs. 0.73 ± 0.09) and RI (0.65 ± 0.06 vs. 0.52 ± 0.07) than the control group (both P<0.001), consistent with previous reports linking increased luteal resistance to luteal dysfunction and threatened abortion (19). In this study, TVCDS exhibited high diagnostic value for threatened abortion, with a sensitivity of 92.86%, specificity of 100%, and AUC of 0.912. Pearson correlation analysis showed that PI and RI were negatively correlated with β-HCG (r=-0.401, -0.465, respectively) and positively correlated with CA125 (r=0.511, 0.492, all P<0.01). The multivariate logistic regression model incorporating PI, β-HCG, and CA125 yielded a combined predicted probability with an AUC of 0.931, superior to any single parameter (P<0.001). These findings validate the combined use of TVCDS and serum markers for early diagnosis and outcome prediction in threatened abortion.

The most important finding of this study is that combining TVCDS parameters with serum biomarkers using a multivariate logistic regression model significantly improves the prediction of failed pregnancy preservation. While individual indicators—PI (AUC = 0.843), β-HCG (AUC = 0.853), RI (AUC = 0.787), and CA125 (AUC = 0.679)—showed only moderate discriminative ability, the combined predicted probability (PRE_1) derived from the model achieved an AUC of 0.931 (95% CI: 0.882–0.980), which was significantly superior to any single parameter (all P<0.01 by DeLong test). At the optimal cutoff of 0.68, the sensitivity and specificity were 86.7% and 88.2%, respectively. Moreover, unlike a simple serial or parallel combination of tests, the model-based PRE_1 provides a continuous risk estimate (0 to 1) rather than a binary classification, allowing clinicians to individualize decision-making. For example, a patient with PRE_1≥0.68 has an approximately 86.7% probability of failed preservation, warranting cautious counseling and avoidance of unnecessary aggressive tocolytic interventions; conversely, those with low PRE_1 may be candidates for targeted progesterone supplementation. This model-based approach thus offers superior clinical utility compared to single markers or non-weighted combinations.

Compared to previous studies, the differentiated innovation of this research is reflected in three respects: First, while numerous studies have investigated the diagnostic value of ultrasound or serum markers individually for threatened abortion, few have systematically evaluated the combined predictive model incorporating corpus luteum Doppler parameters and serum biomarkers using multivariate logistic regression (23, 24). Second, this study quantifies the independent contributions of PI, RI, β-HCG, and CA125 to adverse pregnancy outcomes, providing a clinically applicable prediction tool. Third, it emphasizes the specific correlation between corpus luteum blood flow parameters and CA125, filling a gap in previous research that focused solely on diagnostic efficiency without exploring correlation mechanisms or constructing multivariate predictive models.

Findings in our study define a clear clinical application. The first is the target population: Pregnant women at 6–8 weeks of gestation with vaginal bleeding but no clear gestational sac abnormalities on ultrasound. The second is decision-making guidance: Clinicians can consider corpus luteum dysfunction as a possible cause (indicated by elevated PI/RI) to avoid indiscriminate pregnancy preservation—although direct progesterone measurement was not performed in this study, which should be addressed in future research. Meanwhile, in the case of a low-risk, targeted treatments (e.g., progesterone supplementation) can be provided to improve the success rate of pregnancy preservation. Collectively, this study validates the diagnostic value of TVCDS combined with serum markers for threatened abortion, and assesses pregnancy preservation outcomes, which may facilitate clinical practice and guide clinic treatment. However, this study still has several limitations that can be addressed in future research: ① Single-center-sourced sample: This was a retrospective and single-center study with limited sample extrapolation. Multi-center, prospective studies with larger samples from various regions can be conducted in the future to improve reliability; ② Lack of high-risk populations: This study did not enroll patients with recurrent miscarriages or thyroid dysfunction. In the future, diagnostic threshold adjustments for combined indicators can be explored in high-risk subgroups to improve clinical applicability; ③ Potential measurement errors: Two-dimensional ultrasound measurements of blood flow parameters may be subject to angles. Therefore, three-dimensional ultrasound or elastography techniques can be adopted in the future, coupled with inter-sonographer consistency testing (Kappa=0.89 in this study, which can be further validated by expanding the sample size) to quantify the measurement reliability. ④ Potential inclusion of uterine anomalies: although uterine malformations were set as exclusion criteria, four cases were identified post-enrollment. Sensitivity analysis excluding these cases yielded consistent results, suggesting that their inclusion did not bias the main findings. Future studies should incorporate detailed baseline uterine imaging to further minimize this confounder. ⑤Restriction to primiparous women: Our study population was limited to primiparous women to reduce confounding, which may limit generalizability to multiparous populations. In clinical practice, multiparous women with suspected corpus luteum dysfunction may be more likely to receive targeted progesterone therapy. Future studies should include multiparous women to validate the predictive utility of combined indicators in this population.

## 5 Conclusion

In conclusion, TVCDS combined with serum β-HCG and CA125 has significant value in the early diagnosis of threatened abortion and prediction of adverse pregnancy outcomes. Multivariate logistic regression identified PI, β-HCG, and CA125 as independent predictors. The combined model using predicted probability provides superior and individualized risk assessment compared to any single parameter. These findings may guide clinical decision-making and risk stratification.

## Data Availability

The original contributions presented in the study are included in the article/supplementary material. Further inquiries can be directed to the corresponding author.

## References

[1] Lin L Feng L Tao Y Xing XH Fang LN Zhang HB . Clinical application value of transvaginal detection in diagnosis of early pregnancy abortion. China Digital Med. (2020) 15:142–4:141. doi: 10.3969/j.issn.1673-7571.2020.05.049

[2] Ma Q Lin L Liang XJ Sun FQ . Significance of color doppler ultrasonography in monitoring uterine artery hemodynamic parameters of pregnant women with gestational hypertension and its effect on maternal and infant outcomes. Chin J Reprod Health. (2019) 30:524–9:601. doi: 10.3969/j.issn.1671-878X.2019.06.006

[3] Cao J . Value of transvaginal color doppler ultrasonography in differential diagnosis of threatened abortion in pregnant women in early pregnancy. J Clin Med Pract. (2020) 24:94–6. doi: 10.7619/jcmp.202009027

[4] Xie X Kong B Duan T . Obstetrics and Gynecology. Beijing: People's Medical Publishing House (2018). M.

[5] Aktemur G Tokgöz Çakır B Sucu S Karabay G Şeyhanlı Z Vanlı Tonyalı N . Does corpus luteum doppler have a role in prognostic prediction for outcome with threatened abortion? J Clin Med. (2025) 14:1419. doi: 10.3390/jcm14051419 40094848 PMC11899976

[6] Tuuli MG Norman SM Odibo AO Macones GA Cahill AG . Perinatal outcomes in women with subchorionic hematoma: a systematic review and meta-analysis. Obstet Gynecol. (2011) 117:1205–12. doi: 10.1097/AOG.0b013e31821568de 21508763

[7] Abdallah Y Daemen A Guha S Syed S Naji O Pexsters A . Gestational sac and embryonic growth are not useful as criteria to define miscarriage: a multicenter observational study. Ultrasound Obstet Gynecol. (2011) 38:503–9. doi: 10.1002/uog.10075 21858883

[8] Li KD . Analysis of value of transvaginal color doppler ultrasound combined with serum E2 and β-HCG in the early prediction of pregnancy outcomes. Shaanxi Med J. (2020) 49:732–5. doi: 10.3969/j.issn.1000-7377.2020.06.023

[9] Kim CR Lavelanet AF Ganatra B . Enabling access to quality abortion care: WHO's abortion care guideline. Lancet Glob Health. (2022) 10:e467–8. doi: 10.1016/S2214-109X(21)00552-0 35303448 PMC8938763

[10] Liu Y Lv W . The diagnostic value of transvaginal color doppler ultrasonography plus serum β-HCG dynamic monitoring in intrauterine residue after medical abortion. Med (Baltimore). (2023) 102:e31217. doi: 10.1097/MD.0000000000031217 36749252 PMC9901960

[11] Tsakiridis I Bousi V Dagklis T Sardeli C Nikolopoulou V Papazisis G . Epidemiology of antenatal depression among women with high-risk pregnancies due to obstetric complications: a scoping review. Arch Gynecol Obstet. (2019) 300:849–59. doi: 10.1007/s00404-019-05270-1 31422459

[12] Glicksman R McLeod SL Thomas J Varner C . Services for emergency department patients experiencing early pregnancy complications: a survey of Ontario hospitals. CJEM. (2019) 21:653–8. doi: 10.1017/cem.2019.344 31196232

[13] Ndembi Ndembi AP Mekuí J Pheterson G Alblas M . Midwives and post-abortion care in Gabon: "Things have really changed. Health Hum Rights. (2019) 21:145–55. PMC6927380 31885444

[14] Karataşlı V Kanmaz AG İnan AH Budak A Beyan E . Maternal and neonatal outcomes of adolescent pregnancy. J Gynecol Obstet Hum Reprod. (2019) 48:347–50. doi: 10.1016/j.jogoh.2019.02.011 30794955

[15] Kanmaz AG İnan AH Beyan E Budak A . The effects of threatened abortions on pregnancy outcomes. Ginekol Pol. (2019) 90:195–200. doi: 10.5603/GP.a2019.0035 30901073

[16] Casas X Kimathi-Osiemo M Redwine D Tebbets C Plafker K . Preventing state harassment of abortion providers: the work of the legal support network in Latin America and East Africa. Health Hum Rights. (2019) 21:181–7. PMC6927384 31885447

[17] Salomon LJ Alfirevic Z Bilardo CM Chalouhi GE Ghi T Kagan KO . ISUOG practice guidelines: performance of first-trimester fetal ultrasound scan. Ultrasound Obstet Gynecol. (2013) 41:102–13. doi: 10.1002/uog.12342 23280739

[18] Wu T Lin B Li K Ye J Wu R . Diagnosis and treatment of uterine artery pseudoaneurysm: case series and literature review. Med (Baltimore). (2021) 100:e28093. doi: 10.1097/MD.0000000000028093 34941050 PMC8702271

[19] Vogel JP Vannevel V Robbers G Gwako G Lavin T Adanikin A . Prevalence of abnormal umbilical arterial flow on doppler ultrasound in low-risk and unselected pregnant women: a systematic review. Reprod Health. (2021) 18:38. doi: 10.1186/s12978-021-01088-w 33579315 PMC7881445

[20] Liu Z Feng Y Qin X . A comparative study of transabdominal ultrasound and transvaginal color doppler ultrasonography in the diagnosis of ectopic pregnancy. J Imaging Res Med. (2025) 9:56–8:62. doi: 10.20267/j.issn.2096-3807.2025.16.016

[21] Aksin S Andan C Tunc S Goklu MR . Comparison of brachial artery flow-mediated dilatation, uterine artery doppler, and umbilical artery doppler measurements in obese and normal pregnant women. J Obstet Gynaecol Res. (2022) 48:340–50. doi: 10.1111/jog.15092 34729866

[22] Alsharqi M Ismavel VA Arnold L Choudhury SS Solomi VC Rao SRS . Focused cardiac ultrasound to guide the diagnosis of heart failure in pregnant women in India. J Am Soc Echocardiogr. (2022) 35:1281–94. doi: 10.1016/j.echo.2022.07.014 35934263

[23] De Oliveira Carniello M Oliveira Brito LG Sarian LO Bennini JR . Diagnosis of placenta accreta spectrum in high-risk women using ultrasonography or magnetic resonance imaging: systematic review and meta-analysis. Ultrasound Obstet Gynecol. (2022) 59:428–36. doi: 10.1002/uog.24861 35041250

[24] Menekse Beser D Oluklu D Uyan Hendem D Yildirim M Serbetci H Kara O . Assessment of fetal pulmonary artery doppler indices in pregnant women with rheumatoid arthritis and ankylosing spondylitis. J Clin Ultrasound. (2023) 51:983–91. doi: 10.1002/jcu.23471 37119433

